# Application of Electric Cell-Substrate Impedance Sensing to Investigate the Cytotoxic Effects of Andrographolide on U-87 MG Glioblastoma Cell Migration and Apoptosis

**DOI:** 10.3390/s19102275

**Published:** 2019-05-16

**Authors:** Sheng-Po Chiu, Buyandelger Batsaikhan, Huei-Mei Huang, Jia-Yi Wang

**Affiliations:** 1 Graduate Institute of Medical Sciences, College of Medicine, Taipei Medical University, Taipei 11031, Taiwan; shengpo.chiu@gmail.com (S.-P.C.); buyndlgr@gmail.com (B.B.); cmbhhm@tmu.edu.tw (H.-M.H.); 2 Division of Endocrinology and Metabolism, Department of Internal Medicine, Tri-Service General Hospital Songshan Branch, National Defense Medical Center, Taipei 10581, Taiwan; 3 Department of Physiology, School of Medicine, College of Medicine, Taipei Medical University, Taipei 11031, Taiwan

**Keywords:** ECIS, andrographolide, temozolomide, suberoylanilide hydroxamic acid, glioblastoma multiforme

## Abstract

Glioblastoma multiforme (GBM) is the most common and aggressive primary brain tumor in adults. In recent studies, the efficacy of suberoylanilide hydroxamic acid (SAHA) has been investigated for GBM. We explored the effects of two exploratory compounds, the histone deacetylase SAHA and the natural product andrographolide, on Uppsala 87 Malignant Glioma (U-87 MG) cell migration and viability in comparison with the clinically used therapeutic agent temozolomide (TMZ). We used the electric cell–substrate impedance sensing (ECIS) system to monitor the migration of U-87 MG cells after treatment with various concentrations of these compounds. Moreover, we used the Alamar blue assay and western blotting to observe the concentration-dependent changes in the viability and apoptosis of U-87 MG cells. Our results demonstrated that both SAHA and andrographolide (10–300 μM) significantly inhibited GBM cell migration in a concentration-dependent manner, and 10 μM SAHA and 56 μM andrographolide demonstrated remarkable inhibitory effects on U-87 MG migration. Western blotting indicated that compared with TMZ, both SAHA and andrographolide induced higher expression levels of apoptosis-related proteins, such as caspase-3, BAX, and PARP in U-87 MG cells. Furthermore, all three drugs downregulated the expression of the antiapoptotic protein Bcl-2. In conclusion, SAHA and andrographolide showed exceptional results in inhibiting cell migration and motility. The ECIS wound healing assay is a powerful technique to identify and screen potential therapeutic agents that can inhibit cancer cell migration.

## 1. Introduction

Glioblastoma multiforme (GBM) is the most common and aggressive type of brain tumor in humans [1]. Among all cancers, it is one of the most challenging malignancies to treat in oncology [2]. Patients with GBM have an almost 100% mortality rate with a median survival of 14 months despite highly aggressive regimens consisting of maximal surgical resection, external beam radiation therapy, and adjuvant temozolomide (TMZ) chemotherapy. The very high mortality rate is due to the rapid proliferation and invasion of GBM and its resistance to conventional cancer therapy. Surgical resection followed by chemoradiation therapy has limited efficacy because of poor blood–brain barrier (BBB) penetration, intrinsic GBM resistance, and chemotherapeutic agent toxicity [1,3,4]. More aggressive radiotherapy improves patient outcomes and overall survival; however, the risk of radiation-related complications also increases [5]. Conventional chemotherapy with TMZ is used because it induces considerable DNA damage and triggers the apoptosis of glioma cells [6]. However, clinical toxicity is observed in almost 20% of patients treated with TMZ, and improved survival of 2 years is observed in 27% of patients treated with TMZ; glioma cells develop resistance quickly and at a high frequency to TMZ [7,8,9,10]. Therefore, a novel therapeutic agent is needed. Histone deacetylase (HDAC) inhibitors are considered among the most promising types of therapeutic agents because of their multiple mechanisms for promoting tumor cell death [11].

The most advanced HDAC inhibitor to be investigated in clinical trials for GBM is suberoylanilide hydroxamic acid (SAHA) [12]. SAHA has several anticancer effects such as induction of apoptosis and growth arrest. Another new drug with potent anti-inflammatory and anticancer effects, such as inhibition of invasion and migration, is andrographolide, an active diterpenoid compound [10,13,14] that is extracted from the leaves and stem of *Andrographis paniculata*. Because of its high lipid solubility, it can permeate the BBB; thus, it is a promising agent for targeting glioblastomas. Its pharmacological activities include anti-inflammatory effects generally and anticancer effects particularly. Furthermore, studies have reported andrographolide suppresses cell motility and proliferation in GBM8401, U251, U257, and U-87 cells through the induction of G2/M arrest [15,16,17,18]. Substantial research on andrographolide has revealed that it plays a considerable role in apoptosis through various signaling pathways [19,20,21]. As a natural drug, andrographolide shows reduced cytotoxicity and significantly inhibits tumor growth [15,18]. Moreover, tests in animal models have revealed that it is nearly nontoxic [22].

Migration is a fundamental property of most living cells, and numerous cellular activities of normal and cancer cells involve migration. Cell migration involves a complex series of events implemented in a coordinated and integrated manner, such as protrusion, adhesion, traction, and tail retraction. Exposure to a cytotoxic compound may exert effects at any point in this cascade of events and alter cell migration behavior. Cell migration is measured using conventional methods, such as microscopic observations of cells aided with time-lapse cameras; cell migration is difficult to quantify, and the conventional measurement methods usually requires image processing coupled with extensive data manipulation. In this study, we investigated the cytotoxic effects of TMZ, SAHA, and andrographolide on migration of human glioblastoma Uppsala 87 Malignant Glioma (U-87 MG) cells through a label-free and noninvasive method, namely electric cell–substrate impedance sensing (ECIS). The basic principle of ECIS is to monitor the changes in the electrical impedance of adherent cells grown on gold film electrodes; an ECIS system comprises a small sensing electrode and a large counter electrode, and culture medium is used as the electrolyte. The ECIS method has been used extensively in cancer research and in studies to monitor drug-induced cellular effects for drug discovery, but it has not been used in U-87 MG cells [23,24,25,26]. In the present study, ECIS wound healing assay was applied to evaluate the effects of TMZ, SAHA, and andrographolide on the migration of U-87 MG cells. Western blot analysis was also performed to examine the expression of apoptosis-related proteins and confirm the effects of these drugs on the apoptotic pathway in U-87 MG cells.

## 2. Materials and Methods

### 2.1. Cell Culture and Drug Treatment

The human primary glioblastoma cell line Uppsala 87 Malignant Glioma (U-87 MG, ATCC^®^ HTB-14™) cells were obtained from Invitrogen (Carlsbad, CA, USA). U-87 MG were cultured at 37 °C and 5% CO_2_ in Eagle’s minimum essential medium (ATCC 30-2003) supplemented with the following: 10% fetal bovine serum, 100 μg/mL streptomycin, and 100 units/mL penicillin [8]. Cells were subcultured when they reached 80% confluence, and the medium was changed every 48 h. All cells were grown in culture dishes prior to seeding into ECIS electrode wells. TMZ and SAHA were dissolved in dimethyl sulfoxide (DMSO) and were diluted in cell culture medium to final concentrations of 10, 30, 100, and 300 μM. Andrographolide was similarly prepared to final concentrations of 10, 30, 56, and 100 μM; 0.5% DMSO was used as control.

### 2.2. Impedance Measurement

The ECIS ZTheta 16W components and instrument were obtained from Applied BioPhysics (Troy, NY, USA). Each 8W1E electrode Each electrode array consisted of eight wells with a height of 1 cm and with a bottom area of 0.8 cm^2^; each array consisted of a gold sensing electrode with a diameter of 250 μm and an area of ~5 × 10^−4^ cm^2^ and a much larger gold counter-electrode. The electrodes were connected through a relay bank to a phase-sensitive lock-in amplifier, which measured in- (real) and out-of-phase (imaginary) voltages across the cell-covered electrode. A computer converted these voltages into resistive and capacitive components of complex impedance. The instrument recorded time-series impedance data at multiple frequencies ranging from 62.5 Hz to 64 kHz. Cells were seeded onto the electrodes at a density of 9 × 10^4^ cells/cm^2^ and were allowed to attach and spread for 24 h before drug treatment.

### 2.3. ECIS Wound Healing Assay

For the wound healing assay, cells were grown to confluence for 24 h in the ECIS wells and treated with various concentrations of drugs for another 24 h. The ECIS system can achieve a higher AC voltage (from 100 mV to 3 V) through a 1-kΩ resistor and can thus induce an elevated current (0.1 mA to 3 mA) to wound cells on the sensing electrodes. In this study, an elevated current pulse of 2 mA at 40 kHz was applied for 15 s to electrically wound U-87 MG cells, leading to the death and detachment of cells. The impedance decreased to that of a cell-free electrode, indicating that the desired wounding effect was achieved. Following the wounding process, the living cells surrounding the sensing electrode, which had not been subjected to the elevated current pulse, migrated inward to replace the killed cells until the wound was closed by these cells. The ECIS system subsequently switched back to a normal status, and migration under various experimental conditions were monitored through continuous impedance measurements for at least 10 h. To quantify the wound healing process, the measured resistance data at 4 kHz were selected at 10 h post the wounding process, and the slope was fitted using a linear equation from baseline to plateau. The half-time, T50, was defined as the half-way recovery time along the slope. The wound recovery rate was inversely proportional to T50.

### 2.4. Alamar Blue Cell Viability Test

An Alamar blue cell viability reagent was used to assess cell viability. In two 96-well plates, U87-MG cells were exposed to andrographolide and then incubated with the cell viability reagent was added into two different 96-well plates with the U87-MG cells for 4 h incubation at 37 °C. All fluorescence signals of the 96-well plates were measured. All data were calculated using the following equation:

$$\mathrm{Reduction}\;\left(\%\right)=\frac{\mathrm{Experimental}\;\mathrm{RFU}\;\mathrm{value}-\mathrm{Negative}\;\mathrm{control}\;\mathrm{RFU}\;\mathrm{value}}{100\%\;\mathrm{reduced}\;\mathrm{positive}\;\mathrm{control}\;\mathrm{RFU}\;\mathrm{value}-\mathrm{Negative}\;\mathrm{control}\;\mathrm{RFU}\;\mathrm{value}}\times 100$$

### 2.5. Western Blotting

Total proteins were extracted from U87-MG cells using RIPA buffer (25 mM Tris-HCl, pH 7.6; 150 mM NaCl; 1% NP-40; 1% sodium deoxycholate; and 0.1% SDS) containing a protease inhibitor cocktail (complete, Mini, EDTA-free, Roche Applied Science, Penzberg, Germany) and homogenized using an ultrasonic homogenizer. After quantitative analysis using the Bradford protein assay with a Bio-Rad Dye Reagent (500-0006, Bio-Rad, Hercules, CA, USA), the protein samples were denatured with 5× sample buffer (10% SDS, 25% beta-mercaptoethanol, 50% glycerol, 0.25 M Tris-HCl [pH 6.8], and 0.01% bromophenol blue) and incubated in a water bath at 95°C for 10 min. Cell protein lysates were loaded into 10% SDS-polyacrylamide gels. The gels were transferred onto PVDF filters and incubated with a specific primary antibody to caspase-3 (1:1000) (Novusbio Inc., USA), cleaved caspase-3 (1:1000) (Novusbio Inc., Centennial, CO, USA), PARP (1: 1000) (Cell Signaling Inc., Danvers, MA USA), cleaved PARP (1:1000) (Cell Signaling Inc.), and β-actin (1:1000) (Cell Signaling Inc.). All blots were normalized to the internal standard of β-actin. Bands of interest were visualized using ECL reagents (PerkinElmer, Waltham, MA, USA) and quantified using the UVP BioImaging system (Biospectrum AC Imaging System, Upland, CA, USA) and the ImageJ software program (National Institutes of Health, Bethesda, MD, USA).

### 2.6. Statistical Analysis

Statistical analysis was performed using the Student’s *t* test and one-way ANOVA. The level of significance was set at * *p* < 0.05 and ^+^ *p* < 0.05. All data are expressed as mean ± standard deviation and means ± standard error mean.

## 3. Results

### 3.1. Cell Morphology

**Figure 1:**
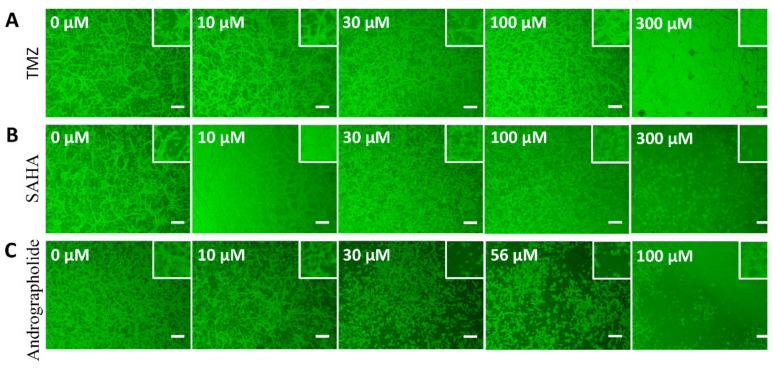
Cytotoxic effects of drug treatment on U-87 MG cells. Phase-contrast images reveal cell morphology at 24 h after drug induction and are compared with those of drug-free cell controls. (**A**) Treatment with 10, 30, 100, and 300 µM TMZ; (**B**) 10, 30, 100, and 300 µM SAHA; (**C**) 10, 30, 56, and 100 µM andrographolide. A concentration-dependent decrease was observed after cells were treated with a higher concentration of each drug. Scale bar = 200 µm.

Figure 1 presents the phase-contrast images of confluent U-87 MG cells treated with various concentrations of TMZ, SAHA, and andrographolide for 24 h. Cells displayed shrunken morphology and other gross features after their exposure to 300 μM TMZ, 30 μM SAHA, or 30 μM andrographolide. These cytotoxic responses, including the decrease in adherent cell number and the increase in cell clumps, were even noticeable when U-87 MG cells were exposed to higher concentrations (>30 μM) of SAHA or andrographolide.

### 3.2. Cell Viability

**Figure 2:**
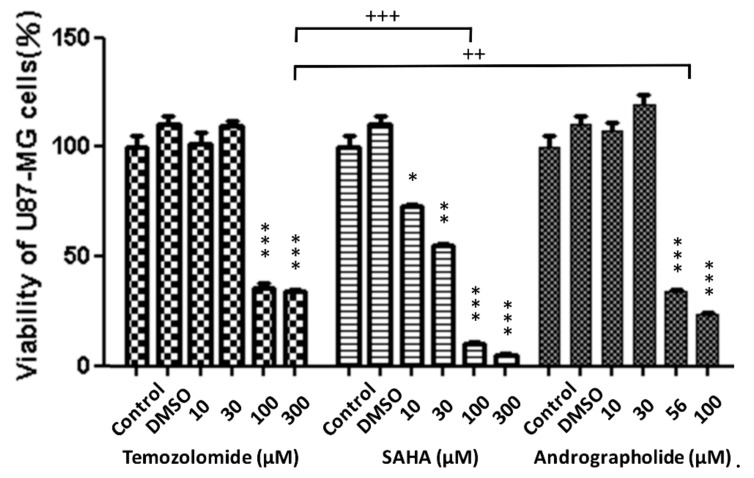
Effects of TMZ, SAHA, and andrographolide on cell viability. Cell viability of U-87 MG cells cultured in 96-well plates under the effect of 10–300 μM TMZ, SAHA, and andrographolide for 24 h compared with cells without drugs and with DMSO. Cells were analyzed using the Alamar blue cell viability assay. Results are expressed as mean ± standard error. *versus control. * *p* < 0.05, ** *p* < 0.01, *** *p* < 0.001, ++ *p* < 0.01, +++ *p* < 0.001.

The cytotoxicity of 10–300 μM TMZ and SAHA and 10–100 μM andrographolide was evaluated using the Alamar blue cell viability assay. As illustrated in Figure 2, cell viability in the control group and in the DMSO group were maintained the same level without change in all three drug classes. At the highest concentrations of 100–300 μM, a dramatic decrease was noted in cell viability in all three drug classes. At lower concentrations of 10–30 μM, the TMZ and andrographolide groups displayed slight variability compared with the control and DMSO groups. At the lower concentrations, the SAHA group displayed a 30–40% decrease in cell viability.

### 3.3. Real-Time Monitoring of U-87 MG Cell Attachment and Spreading

**Figure 3:**
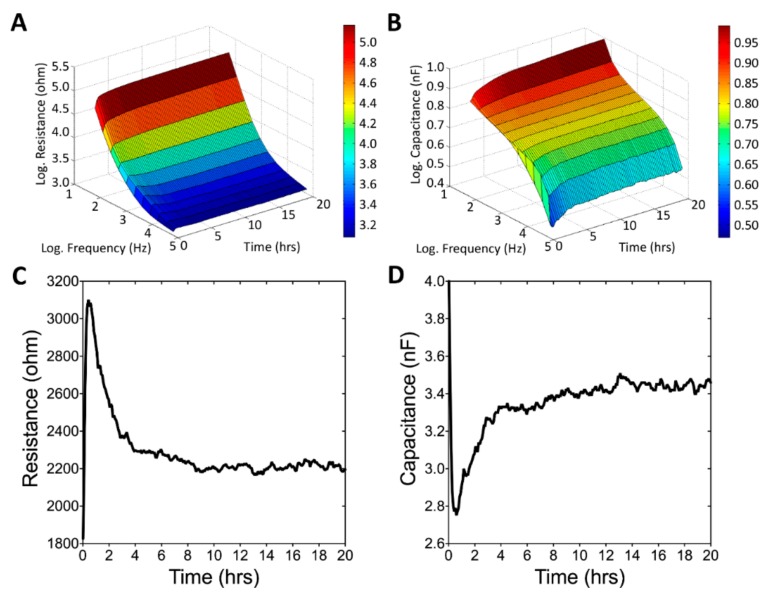
ECIS measurement of U-87 MG cell attachment and spreading. At time zero, U-87 MG cells were seeded in an ECIS well giving a final cell density of 9E4 cells per cm^2^. Three-dimensional representation of the changes in resistance (**A**) and capacitance (**B**) as a function of frequency and time during the attachment and spreading of U-87 MG cells on microelectrodes. (**C**) Changes in resistance as a function of time measured at 4 kHz. (**D**) Changes in capacitance as a function of time measured at 64 kHz.

**Figure 4:**
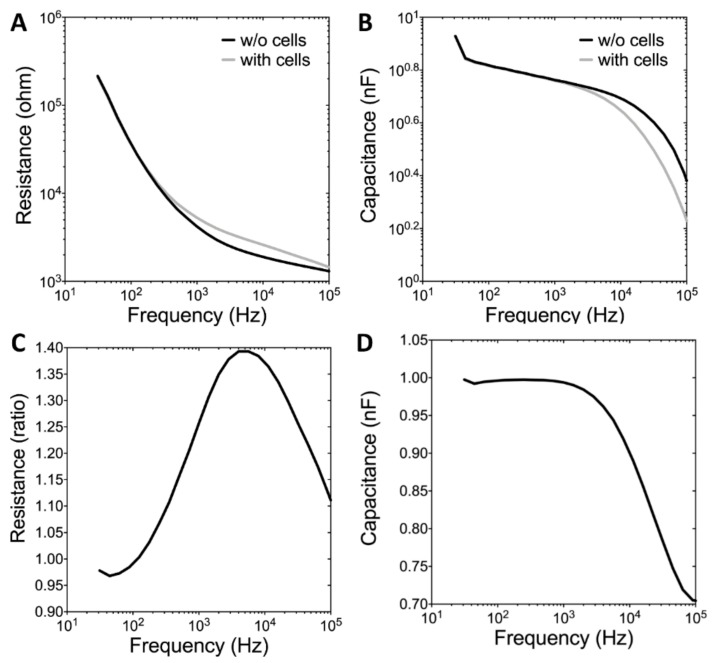
(**A**) Resistance data and (**B**) capacitance data as a function of frequency obtained from cell-free (dotted line) and cell-covered (solid line) electrodes. (**C**) Normalized resistance and (**D**) normalized capacitance obtained from electrodes confluent with U-87 MG cells by dividing with the corresponding quantities for the cell-free electrodes.

Figure 3A,B illustrate the long-term monitoring of U-87 MG cell attachment and spreading from the inoculation period to 20 h after cell seeding. Impedance measurements were performed at 11 different frequencies (62.5 Hz–64 kHz). The data obtained from a typical run are presented as three-dimensional graphs to indicate the changes in resistance and capacitance as a function of frequency and time. Because U-87 MG cells cannot grow as a confluent monolayer, the measured impedance of the cell-covered electrode was relatively low, regardless of the frequencies applied here. Figure 3C,D depict the changes in resistance and capacitance as a function of time respectively measured at 4 kHz and 64 kHz, which are the optimal detection frequencies for assessing U-87 MG cells. When cells attach and spread on the sensing electrodes, the main current cannot pass through the insulating cell membrane and must flow around the cells. By effectively blocking the area available for the current flow, a large increase occurs in the impedance of the system. Smaller changes in the cell–electrode interaction due to cell motion cause the impedance to fluctuate with time. As illustrated in Figure 4A,B, when comparing the measured resistance and capacitance as a function of frequency between the cell-free and cell-covered electrodes, we know that different cell types have their maximum responses at different frequencies [23,27]. Therefore, when monitoring cellular responses to toxins, the AC signal is usually set at a specific frequency that causes the highest responses to impedance changes caused by cell motion and metabolic activity. Figure 4C,D display both normalized resistance and capacitance as a function of frequency obtained from electrodes confluent with U-87 MG cells by dividing with the corresponding quantities for the cell-free electrodes. The normalized resistance spectra are displayed as biphasic curves, with the peak value at 4 kHz.

### 3.4. Wound Healing and Migration of U-87 MG Cells under Drug Treatment

To determine the effective inhibitory concentrations of TMZ, SAHA, and andrographolide for U-87 MG cell migration, cells were first seeded into electrode-containing wells and allowed to attach and spread for approximately 24 h. Confluent cells were subsequently treated with various concentrations of drugs for another 24 h, and an ECIS wound healing assay was performed. The measured resistance data at 4 kHz were selected at 10 h post the wounding process, and the slope was fitted using a linear equation from baseline to plateau.

**Figure 5:**
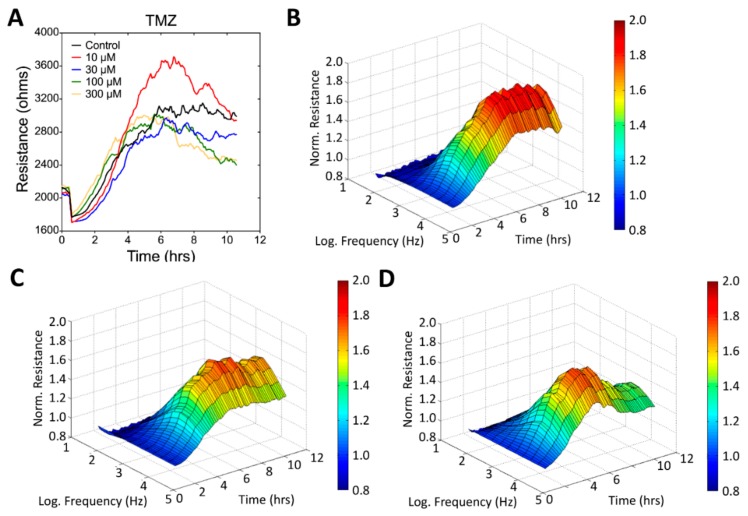
Effects of TMZ on the wound healing and migration of U-87 MG cells measured using ECIS. (**A**) Time course of the measured resistance at an AC frequency of 4 kHz during the ECIS wound healing assay. Three-dimensional demonstration of the effects of TMZ at concentrations of (**B**) 0 μM (control), (**C**) 30 μM, and (**D**) 300 μM on cell migration.

**Figure 6:**
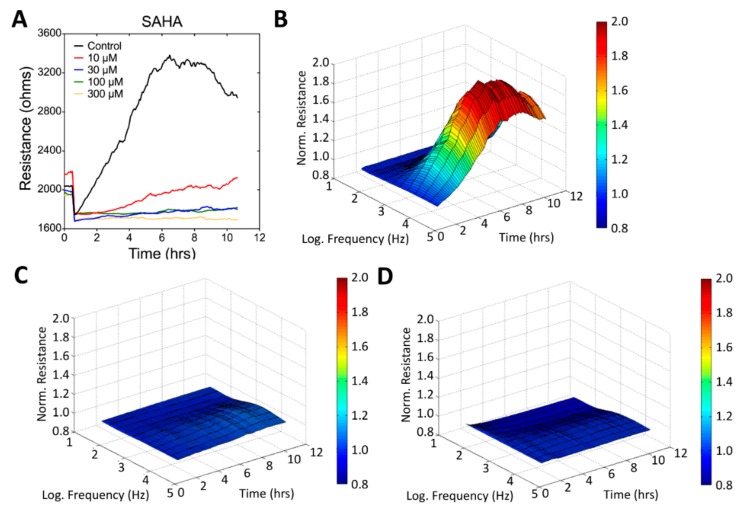
Effects of SAHA on the wound healing and migration of U-87 MG cells measured using ECIS. (**A**) Time course of the measured resistance at 4 kHz; three-dimensional demonstration of the effects of SAHA at concentrations of (**B**) 0 μM (control), (**C**) 30 μM, and (**D**) 300 μM on cell migration.

**Figure 7:**
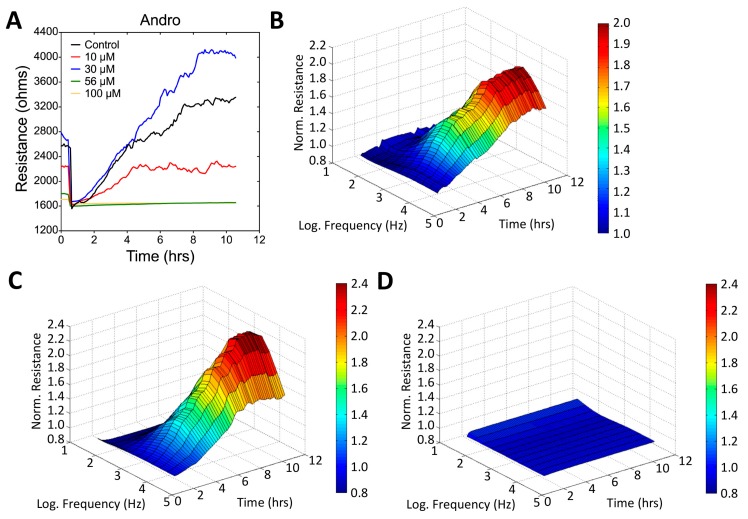
Effects of andrographolide on the wound healing and migration of U-87 MG cells measured using ECIS. (**A**) Time course of the measured resistance at 4 kHz; three-dimensional demonstration of the effects of andrographolide at concentrations of (**B**) 0 μM (control), (**C**) 30 μM, and (**D**) 100 μM on cell migration.

**Figure 8:**
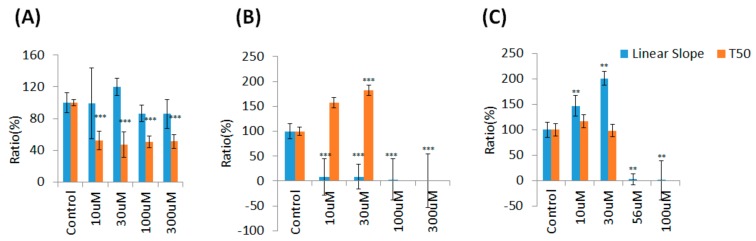
Linear slope and T50 values obtained from the ECIS wound healing assay of U-87 MG cells in response to various concentrations of (**A**) TMZ (*n* = 6), (**B**) SAHA (*n* = 6), and (**C**) andrographolide (*n* = 5). According to the wound recovery data obtained from exposure to 100 μM and 300 μM SAHA and 56 μM and 100 μM SAHA, the linear slope was too low; therefore, T50 was not calculated and presented. Significance was calculated by comparing each experimental group with their control group. ** *p* < 0.01; *** *p* < 0.005.

As illustrated in Figure 5A, cells were electrically wounded at the 0.5 h time point, and data acquisition was briefly suspended for 6 min. After wounding, resistance values after wounding decreased to approximately 1.6–2 kΩ, which is close to the value of a cell-free electrode, indicating cell death and detachment on the sensing electrodes. Data acquisition was then restarted at time point of 0.6 h. Figure 5B–D represent the three-dimensional demonstrations of the normalized resistance as a function of frequency and time. These three-dimensional profiles indicate that the inhibitory effects of TMZ were observed over a wide range of frequencies (from 500 Hz to 64 kHz). For comparison of the results for various TMZ concentrations, the measured resistances at various frequencies were normalized (divided by the value at the time point of 0.6 h) for these three-dimensional data sets. Figure 6 and Figure 7 present the data taken from the inhibitory effects of SAHA and andrographolide respectively. The data are presented in the same way as Figure 5; however, the effects are more noticeable when compared with those of TMZ. To quantitate the wound healing data presented in Figure 5A, the recovery slope was fitted, and T50 was calculated for each TMZ concentration (Figure 8A). No significant difference in the recovery slope was observed for each experimental condition (Figure 8A). Surprisingly, compared with the control group, the values of the half-time (T50) became smaller, indicating a higher wound recovery rate for U-87 MG cells 24 h after treatment with 10–300 μM TMZ (Figure 8A). It is worth noted that the wound healing process, as represented by each resistance time series curves, is not necessary to return to its original value since U-87 MG cells are incapable of forming a confluent monolayer on the sensing electrode. 

Specifically, SAHA at a concentration as low as 10 μM displayed evident inhibitory effects on the migration of U-87 MG cells (Figure 6). As shown in Figure 8B, compared with the control group, U-87 MG cells treated with 10 or 30 μM SAHA showed much lower recovery slopes and much higher T50 values. Interestingly, while U-87 MG cells exposed to 30 μM andrographolide seem to be agitated and to migrate faster, their wound healing and migration were completely inhibited after 24-h exposure to 100 μM (Figure 7 and Figure 8C) or 300 μM andrographolide (data not shown). In order to observe the cytotoxic trend of andrographolide more clearly, cells exposed to 56 μM andrographolide had been further tested and the result also displayed an evidently inhibitory effect on wound healing and migration (Figure 7 and Figure 8C).

### 3.5. Apoptosis-Related Protein Expression in Drug-Treated U-87 MG Cells

**Figure 9:**
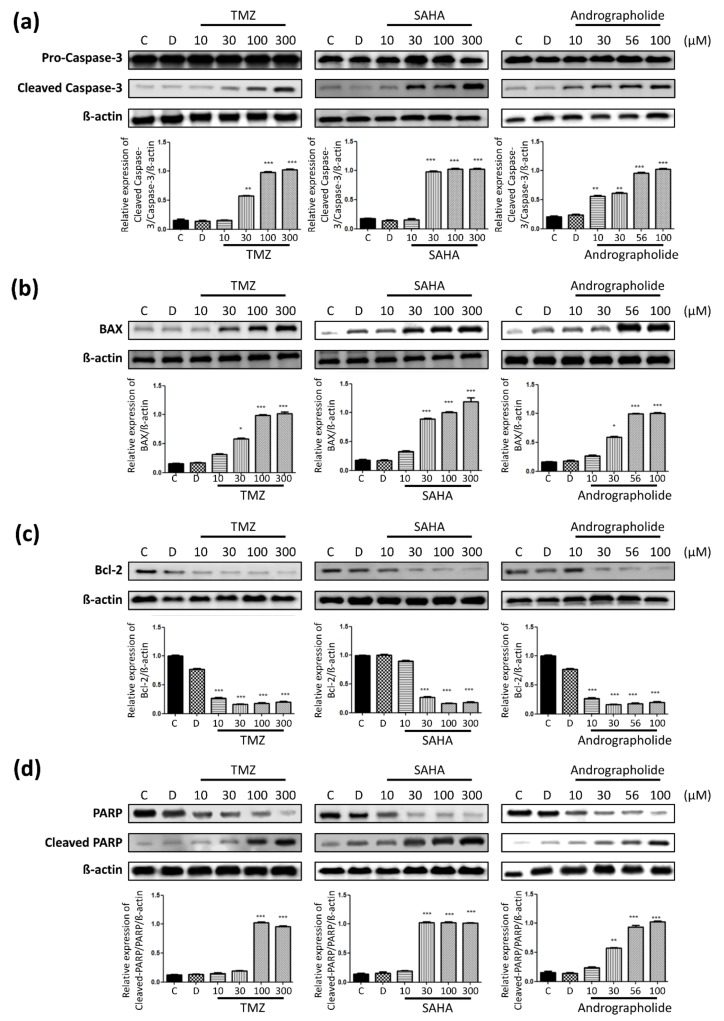
Protein expression in U-87 MG cells after drug treatment. Cells were treated with various concentrations of each drug for 24 h, and western blotting was conducted to analyze protein levels, which were adjusted using β-actin. (**a**) Caspase-3 and cleaved caspase-3. (**b**) BAX. (**c**) Bcl-2. (**d**) PARP and cleaved PARP. The results are expressed as mean ± standard error of the mean from three determinations per condition (*n* = 5). * *p* < 0.05, ** *p* < 0.01, *** *p* < 0.001.

To determine the mechanism through which these drugs induce cell death, we analyzed the expression of several apoptosis-related proteins in U-87 MG cells treated with various concentrations (10–300 μM) of these drugs. Caspase-3, BAX, Bcl-2, and PARP were assayed through Western blot analysis 24 h after drug induction. As indicated in Figure 9a, the caspase-3 protein was accumulated in cells treated with all concentrations of each drug. The cleaved caspase-3 protein was mainly accumulated in cells treated with the higher concentration of each drug. Cleaved caspase-3 accumulation was observed in cells treated with 10–300 μM andrographolide. As shown in Figure 9b, BAX protein was highly expressed in cells treated with 100–300 μM andrographolide. SAHA drug expresses BAX protein at 30 μM concentration, contrary to TMZ and andrographolide. As illustrated in Figure 9c, Bcl-2 protein was expressed in the control and DMSO-treated cells but was not highly expressed in drug-treated U-87 MG cells. Moreover, the expression of the Bcl-2 protein, which inhibits apoptosis, decreased. PARP was expressed in the control and DMSO groups and groups treated with 10 μM of all three drugs. By contrast, cleaved PARP was accumulated only at the higher concentrations as observed in Figure 9d. These results indicate that all three drugs simultaneously inhibited cell viability and induced cell apoptosis at later stages. Treatment with higher drug concentrations reinforced the apoptotic effects, specifically for andrographolide and SAHA; SAHA exerted its inhibitory effects at a lower concentration. Based on the western blot analysis results, andrographolide displayed higher effectiveness.

## 4. Discussion

Because most patients with GBM survive less than a year, these tumors have drawn significant research attention; however, they have evaded increasingly clever and intricate attempts at therapy over the last half-century [2]. Surgical resection used to be the mainstay of treatment for glioma. In the last decade, however, opinion has changed regarding the goal of surgical resection in treating glioma [28]. Although the survival rate for GBM has improved with recent advances in treatment, prognosis remains generally poor [29]. One of the reasons for the resistance of GBM to therapeutic intervention is the complex characteristics of GBM. As the name implies, glioblastoma is multiforme. It is multiforme grossly, showing regions of necrosis and hemorrhage. It is multiforme microscopically, with regions of pseudopalisading necrosis, pleomorphic nuclei and cells, and microvascular proliferation [2].

Despite conventional chemotherapeutic methods and surgical resection, GBM is still associated with high mortality. Because of the invasive and highly proliferative properties of glioma, a novel therapeutic method is required. Studies have demonstrated that patients treated with TMZ, a standard chemotherapeutic drug, exhibit clinical toxicity, limiting its treatment efficiency [7]. Inherent boundaries of the central nervous system, such as the BBB or the blood–cerebrospinal fluid barrier, and a general lack of response to numerous chemotherapeutic agents have led to the development of alternative treatment modalities [30]. In this study, two novel drugs under development were compared with TMZ. Extensive studies have revealed that tumor development and progression in various cancer cell lines can be limited by modulating cell apoptosis or invasion. Furthermore, we know that a hallmark of glioma is in the high resistance against caspase-dependent apoptosis [31,32]. This is the first study where we compare the effects and apoptotic pathway of a novel HDAC inhibitor, SAHA, and a natural product, andrographolide, with TMZ. Previous studies have reported that SAHA inhibits cell viability and induces autophagy, and suppresses tumor growth [33]. Similarly, studies have shown the inhibition of invasion and migration in GBM cells under treatment with andrographolide [18].

In this study, Cell viability of U-87 MG cells assessed by Alamar Blue assay indicated that the significantly inhibitory concentrations of TMZ, SAHA, and andrographolide were as low as 100 μM, 10 μM, and 56 μM respectively (Figure 2). ECIS wound healing assay was further used to monitor drug treated U-87 MG cells at different concentrations. In general, measured resistance values at 4 kHz for confluent fibroblastic cell layers such as WI-38 and WI-38 VA13 are about 4.5 kΩ and 9 kΩ respectively [23]. The overall measured resistance after U-87 MG cell attachment and spreading was only about 2-3 kΩ (Figure 3C). The peak value of the normalized resistance spectrum was about 1.4 (Figure 4C), much smaller than those of WI-38 (peak value ~3) and WI-38 VA13 cells (peak value ~6) [23]. This was because U-87 MG cells attached and spread in a web-like manner, branching out with sparse spaces between the cell-to-cell junction (Figure 1). While the detection sensitivity of the ECIS wound healing assay is dependent on these peak values, a general impedance pattern was successfully observed in the wound healing process of the drug treated glioma cells (Figure 5A, Figure 6A and Figure 7A). The initial resistance drop indicated the wounding effect. Following the wounding phase, subsequent cell migration associated with the healing phase has been monitored and analyzed. The differences observed in the wound healing between the three drugs is associated with the migration rate and inhibition effect of the drugs. Our results demonstrated that the healing migration rate (inversely proportional to T50) of U-87 MG cells was considerably reduced by 10 μM SAHA or 56 μM andrographolide, but not by 10-300 μM TMZ (Figure 8). It has been suggested that IC50 value of TMZ at U-87 glioblastoma cells from cell survival assay after 72 h is about 100-200 μM. It is not clear why U-87 MG cells treated with 10-300 μM TMZ exhibited higher recovery rates (shorter T50 values) after wounding than control group did. A possible answer might be that our wound healing assay was performed 24 h after drug treatment. In this study, the wound healing migration properties were measured and represented independent. Quantitative parameters like linear slope and T_50_ can be further developed as a measure for other drug screening or to test drug effect on different cellular conditions. The ECIS method is a powerful technique to identify and develop potential therapeutic agents that can inhibit cell migration.

In parallel with the monitoring of cell conditions under drug treatment, biochemical assays were performed to prove these findings. A positive correlation was exhibited from the protein results. Standard chemotherapeutic drugs exert their inhibitory effects through the intrinsic apoptotic pathway, which was used as a criterion in this study. The data in this study revealed that drugs affected caspase activity and apoptosis in human glioma cells. The apoptosis-related proteins analyzed were caspase-3, BAX, Bcl-2, and PARP. Caspases are a family of cysteine proteases that are activated during apoptosis. Specifically, caspase-3 plays a vital role in the activation of apoptosis; in a previous study, caspases increased cell death and stimulated apoptosis in mice that expressed them [34]. Furthermore, caspase-3 has an active function in the cleavage of the Bcl-2 protein and other substrates such as Bcl-X_L_, which normally functions to prevent apoptosis in glioma cells [35,36,37,38]. The cleavage of the Bcl-2 protein may promote the further activation of caspases, which in turn promote apoptosis [35]. Caspase cleavage has also been linked to the prevention of homeostatic mechanism from apoptosis; the PARP protein plays a central role in the DNA repair mechanism and is inhibited by caspase cleavage [39,40]. The BAX protein is linked with the mitochondrial function that releases cytochrome c and activates caspases in the cytosol. In this study, caspase-3 and cleaved caspase-3 were measured in all treated and control groups of glioma cells. We demonstrated that caspase-3 and cleaved caspase-3 activity increased by at least two-fold in most drug-treated groups, and increases were notable in andrographolide-treated cells. Furthermore, BAX protein expression was correlated with the activity and expression of caspase-3 in the drug-treated groups. High levels of the antiapoptotic protein Bcl-2 were observed in the control group; by contrast, in the drug-treated groups, its expression decreased to less than half of the expression in the control group. Previous studies have demonstrated that the PARP protein plays a role in the inhibition of TMZ treatment efficacy for glioma cells [41]. Closely observing PARP, we can see that it is expressed at higher levels in the higher concentration of each drug group which correlated with the ECIS results. At higher concentrations, the drug effect started to decay and the homeostatic mechanisms began to be activated in cells. The control group expressed a low level of PARP because they are intact and do not necessitate its function. Taken together, these findings were in agreement with the ECIS impedance results and correlated with the results of the cell viability assay at each concentration.

## 5. Conclusions

In this study, we have tested the effects of TMZ, SAHA, and andrographolide on human glioblastoma cells. The cell viability assay results revealed that the lower concentration of each drug is favored for drug treatment on U-87 MG cells. The ECIS system provides label-free detection of U-87 MG cells under drug treatment and real-time monitoring of various cellular activities, including cell adhesion, spreading, and migration over the period of 72 h. Impedance-based measurement revealed a linear concentration-dependent decrease in U-87 MG cells treated with drug concentrations ranging from 10 μM to 300 μM. The wound healing assay successfully monitored the recovery rate of cells under drug treatment. The results revealed that 10 μM andrographolide induced a preferred effect on U-87 MG cells, by decreasing the recovery rate drastically in comparison to TMZ and SAHA at the lowest concentrations. The higher concentrations did not recover, maintaining a resistance of a cell-free electrode.

Further study of apoptosis-related proteins through western blotting demonstrated that SAHA and andrographolide inhibited cell viability and induced apoptosis at later phases from the 10–30 μM concentrations. The results of this study indicated that the most significant inhibitory effect was observed at concentration 100 μM of the andrographolide drug. Furthermore, this study presented a useful impedance-based method for drug screening and cancer research.
